# Fine Tuning the Cytokine Storm by IFN and IL-10 Following Neurotropic Coronavirus Encephalomyelitis

**DOI:** 10.3389/fimmu.2018.03022

**Published:** 2018-12-20

**Authors:** Carine Savarin, Cornelia C. Bergmann

**Affiliations:** Department of Neuroscience, Cleveland Clinic Foundation, Lerner Research Institute, Cleveland, OH, United States

**Keywords:** central nervous system, viral infection, JHMV, IFNα/β, IFNγ, IL-10, demyelination

## Abstract

The central nervous system (CNS) is vulnerable to several viral infections including herpes viruses, arboviruses and HIV to name a few. While a rapid and effective immune response is essential to limit viral spread and mortality, this anti-viral response needs to be tightly regulated in order to limit immune mediated tissue damage. This balance between effective virus control with limited pathology is especially important due to the highly specialized functions and limited regenerative capacity of neurons, which can be targets of direct virus cytolysis or bystander damage. CNS infection with the neurotropic strain of mouse hepatitis virus (MHV) induces an acute encephalomyelitis associated with focal areas of demyelination, which is sustained during viral persistence. Both innate and adaptive immune cells work in coordination to control virus replication. While type I interferons are essential to limit virus spread associated with early mortality, perforin, and interferon-γ promote further virus clearance in astrocytes/microglia and oligodendrocytes, respectively. Effective control of virus replication is nonetheless associated with tissue damage, characterized by demyelinating lesions. Interestingly, the anti-inflammatory cytokine IL-10 limits expansion of tissue lesions during chronic infection without affecting viral persistence. Thus, effective coordination of pro- and anti-inflammatory cytokines is essential during MHV induced encephalomyelitis in order to protect the host against viral infection at a limited cost.

## Introduction

The central nervous system (CNS) is susceptible to various neurotropic viral infections associated with acute inflammation. Depending on the distinct anatomical regions infected, inflammation is referred to as meningitis (meninges), encephalitis (brain), myelitis (spinal cord), or meningoencephalitis and encephalomyelitis if multiple sites are afflicted (1). Viral meningitis is overall more clinically benign, whereas encephalitis is associated with clinical evidence of neurological dysfunctions, which can range from behavioral changes to seizures and paralysis. Many encephalitic viruses such as insect borne viruses, enteroviruses, and non-endogenous retroviruses can rapidly invade the CNS early following peripheral infection. However, encephalitis caused by members of the herpes viruses, e.g., Herpes Simplex Virus (HSV)-2, cytomegalovirus (CMV), or the polyomavirus John Cunningham virus (JC virus) are more commonly caused by immune suppression allowing re-activation of otherwise controlled chronic or latent peripheral infections and invasion of, or reactivation within the brain, resulting in severe disability and death (2). For example, premature death of multiple sclerosis patients treated with Natalizumab due to JC-virus mediated progressive multifocal leukoencephalopathy emphasizes the importance of CNS immune surveillance to prevent viral recrudescence (3, 4).

As many neurotropic viruses predominantly target highly specialized and/or non-renewable cells controlling cognitive and vital physiological functions, an efficient anti-viral immune response is essential to limit viral CNS dissemination to prevent lethal outcomes. However, the anti-viral immune response needs to be tightly regulated to minimize bystander tissue damage and neurological dysfunction, which can be long term sequela even after virus control (2). Given the limitations in obtaining human CNS samples, several murine models of viral encephalitis provide complementary tools to unravel activation, effector function and regulation of protective immune responses within the CNS; these include Vesicular stomatitis virus (VSV), Sindbis virus, West Nile virus, Theiler's encephalomyelitis virus (TMEV) and mouse hepatitis virus (MHV). This review primarily focuses on encephalomyelitis induced by neurotropic MHV, namely the sublethal glia tropic variant of the John Howard Muller MHV strain, designated v2.2-1, and the non-lethal dual liver and neurotropic MHV-A59 strain (5). Both viruses are characterized by an acute encephalomyelitis which resolves into a persistent infection characterized by demyelination and sustained detection of viral RNA in the absence of infectious virus. As demyelination is immune-mediated and neuronal infection is sparse in the v2.2-1 model, it provides a useful tool to study the dynamics and regulation of antiviral host immune responses associated with ongoing immune-mediated tissue damage balanced by repair during chronic infection.

## Mouse Hepatitis Virus

Mouse hepatitis viruses (MHV), members of the positive-strand RNA enveloped *Coronaviridae*, are natural murine pathogens that infect the liver, gastrointestinal tract and CNS (6, 7). Virus tropism and pathogenesis depends upon virus strains and variants, as well as inoculation route (8). The attenuated MHV-JHM v2.2-1 referred as v2.2-1 from hereon is a monoclonal antibody derived variant of the lethal MHV-JHM strain (9), which has been extensively used to unravel immune correlates of protection and viral-induced demyelination. Upon intracranial infection the MHV-A59 strain is more neuronotropic than v2.2-1, but also infects glia and causes immune mediated demyelination, although clinical disease severity in immune competent adult infected mice is less severe (10). Unless otherwise stated, this review pertains to encephalomyelitis induced by v2.2-1. Following intracranial administration, v2.2-1 infects the ependymal cells lining the ventricles before spreading to microglia, astrocytes, and oligodendrocytes (OLG); neurons are largely spared. Peak virus replication around day (d) 5 post-infection (p.i.) correlates with activation of astrocytes and microglia, disruption of the blood brain barrier (BBB) and CNS recruitment of neutrophils, NK cells and predominantly bone marrow derived monocytes (6, 11). Monocytes and neutrophils enhance BBB disruption (12) and pave the way for infiltration of T and B cells. T cell recruitment is associated with signs of encephalitis observed around d7 p.i. Both CD8 and CD4 T cells are essential for reducing infectious virus below detectable levels 2 weeks p.i. (6, 13). T cell mediated antiviral function also correlates with onset of demyelination, which peaks 2–3 weeks after control of infectious virus. While virus replication is no longer detectable in chronically infected mice, persisting viral RNA remains present in spinal cords at slowly declining levels. Deprivation of local humoral immunity constitutes the only manipulation resulting in reemergence or lack of clearance of infectious v2.2-1 or A59 virus (14), suggesting virus persists in a replication competent form controlled by local Ab (15).

Induction of cytokines and chemokines, as well as CNS recruitment of innate and adaptive immune cells, is highly regulated during neurotropic MHV infection, emphasizing the orchestration of specific functions at times critical to efficiently control infectious various, while restraining subsequent tissue destruction. This review discusses findings from our colleagues and own laboratories on the role of signature cytokines associated with effective, yet dampened anti-viral responses and limited tissue damage with focus on Interferon (IFN)α/β, IFNγ and IL-10.

## Type I IFN: Conductor of the Early Anti-Viral Response

The induction of innate immune responses, including type I IFNs, provides the first critical line of immune defense in stemming viral spread throughout the CNS (16, 17). Although coronaviruses are known to be poor IFNα/β inducers, the importance of IFNα/β signaling following both MHV-A59 and v2.2-1 infection, became apparent following infection of IFNα/β receptor deficient (IFNAR^−/−^) mice. Uncontrolled viral replication, extensive viral dissemination throughout the CNS, and expanded tropism to neurons coincided with rapid mortality (18, 19). Early viral replication also induces cytokines and chemokines, some of which are IFNα/β dependent (20). Together, the early response regulates the adaptive immune response essential for reducing viral replication.

Since the naïve CNS is devoid of plasmacytoid dendritic cells, potent peripheral IFNα/β inducers, IFNα/β production relies on sensing of virus invasion by glial and neuronal cells. Although glia and neurons are known to express pattern recognition receptors (PRRs), which recognize diverse pathogen associated molecular patterns (PAMPs) and endogenous danger signals (DAMPs), the diversity and magnitude varies not only between CNS cell type, but also their regional anatomical localization within the CNS (2, 21–23). While all CNS cell types have been shown to be capable of producing IFNα/β *in vitro*, the ability to induce IFNα/β *in vivo* depends on the specific virus, its replication cycle, cellular tropism and respective repertoire of PRRs and associated signaling factors. The disparities between CNS cells in their ability to produce and respond to IFNα/β *in vivo* have recently been reviewed (20). Our own studies with v2.2-1 revealed that oligodendrocytes (OLG) are poor inducers of IFNα/β relative to microglia consistent with low basal levels and limited diversity of PRRs detecting viral RNAs (24). The low expression of IFNα/β receptor chains further coincides with reduced and delayed expression of interferon sensitive genes (ISG) encoding factors with anti-viral activity, including interferon-induced protein with tetratricopeptide repeats 1 and 2 (Ifit1 and Ifit2). Both their reduced ability to establish an antiviral state and upregulate IFNα/β-induced major histocompatibility complex (MHC) class I presentation components may enhance their propensity to become the predominantly infected glia cells and set the stage for establishment of persistent infection (24, 25).

Cell types, which are not effective initial type I IFN inducers, may nevertheless be protected after inducing ISG, which also include PRRs, in response to IFNα/β produced by heterologous cells. Similar to OLG, lower constitutive PRR, and ISG levels were found in astrocytes relative to microglia. However, studies with MHV-A59 revealed delayed but substantial upregulation of IFNα/β pathway genes within astrocytes following infection (26). Some PRRs, ISGs and IFNα were even expressed at higher levels in astrocytes at d5 p.i. compared to microglia, indicating that astrocytes are critical to the innate antiviral activity through amplification of the IFNα/β response. The importance of IFNα/β signaling within astrocytes was confirmed by uncontrolled viral replication and premature death (1 week p.i.) of mice lacking IFNAR expression specifically on astrocytes (26). However, delayed mortality compared to total IFNAR deficiency indicated that other CNS cells, presumably microglia, contribute early to limiting virus dissemination. Analysis using the v2.2-1 virus will determine whether the astrocytic contribution to IFNAR mediated protection remains similar in a model with sparse astrocyte infection.

Altogether, these data shed light on the individual *in vivo* contribution of glial cells in overall IFNα/β mediated early protection against MHV CNS infection. More studies using conditional ablation of IFNAR and selected ISGs in various encephalitic virus models will be beneficial in unraveling the importance of autocrine and paracrine protective IFNα/β effects on subsequent adaptive responses and potential establishment of cell type specific persistence.

## IFNγ and Perforin: When Adaptive Immunity Takes the Relay

Although innate anti-viral immune responses are critical in containing initial CNS virus spread, virus-specific T cell effector functions are essential to eliminate or reduce infectious virus load during most acute infections (27–29). Importantly, CNS cells appear to shape the adaptive immune response to avert direct T cell cytolytic effector mechanisms, especially targeted to neurons, as recently reviewed by Miller at al. (2). While various mechanisms, including intrinsic deviation from cellular targets of lytic granules, T cell inhibitory molecules, as well as anti-inflammatory factors have been demonstrated to dampen T cell effector functions, the same mechanisms also favor establishment of persistent infection.

The requirement for adaptive immune responses to control neurotropic MHV was evidenced by uncontrolled viral replication and mortality of v2.2-1 infected immunodeficient Rag2^−/−^ or SCID mice (30, 31). However, the absence of adaptive immunity also revealed that virus itself does not cause demyelination (6, 9, 32), supporting T cell effector function in mediating pathology. T cell depletion studies subsequently revealed that v2.2-1 control required both CD4^+^ and CD8^+^ T cells, with CD4^+^ T cells providing helper function for CD8^+^ T cells, which are the primary effector T cells within the CNS (13, 33). Efforts to define prominent anti-viral effector function further demonstrated that mice deficient in perforin-mediated cytolysis could not control viral replication in microglia and astrocytes, while virus control in oligodendrocytes (OLG) was unaffected (34). In contrast, IFNγ^−/−^ mice exhibited loss of viral control specifically in OLG (35). The requirement for IFNγ mediated control in OLG was further confirmed by specifically abrogating IFNγ receptor signaling in OLG (36). These data thus demonstrated that T cell mechanisms affecting viral control *in vivo* were clearly cell type dependent, although CD8^+^ T cells isolated from the infected CNS exerted both potent cytolytic activity and produced IFNγ *ex vivo*. The distinct susceptibilities of glia cells to CD8^+^ T cell effector functions was further confirmed by adoptive transfer of virus-specific CD8^+^ T cells deficient in either IFNγ or perforin into infected T cell-deficient mice (13, 31). The overall higher dependency on IFNγ for MHV control may also reside in the differential dependence of glia on IFNγ to upregulate MHC class I and antigen processing components. Whereas, class I surface expression by microglia coincides with IFNα/β expression, OLG appear to require IFNγ to upregulate class I (25). This delayed class I expression coinciding with enhanced expression of the inhibitory receptor B7-H1 may protect OLG from CD8^+^ T cell cytolysis (37).

Analysis of the relative contribution of CD8^+^ vs. CD4^+^ T cells to express IFNγ following v2.2-1 infection surprisingly revealed that CD4^+^ T cell express higher levels of IFNγ mRNA at the population levels than CD8^+^ T cells (38). However, the APC triggering IFNγ production by CD4^+^ T cells have not been identified, but may be meningeal or perivascular DC. CD4^+^ T cells can indeed mediate direct anti-viral activity in addition to enhancing CD8^+^ T cell migration and survival within the CNS (39). However, adoptive transfer of perforin- or IFNγ-deficient CD4^+^ T cells into infected immunodeficient recipients revealed that viral control was independent of either anti-viral function (13, 17). Moreover, sparse MHC class II upregulation on microglia in the absence of IFNγ, and lack of MHC class II expression on astrocytes and OLG suggest that CD4^+^ T cells contribute to viral control indirectly via a viral antigen cross presenting APC or via an MHC class II-independent mechanisms (17). Cell types presenting viral antigen to activate CD4^+^ T or CD8^+^ T cells in the CNS *in vivo* requires more extensive investigation not only in the MHV model, but also models of neuronotropic infection.

Although the anti-viral T cell response is vital to protect the host following neurotropic infection, it induces tissue damage characterized by demyelination and modest axonal damage. A role for cytolytic infection of OLG was discounted based on the lack of tissue damage in immunodeficient mice, as well as restored myelin loss by transfer of virus specific CD4^+^ or CD8^+^ T cells (7). Direct T cell-mediated cytolysis of OLG is also unlikely given the IFNγ dependent control of infectious virus and difficulties to detect apoptotic OLG (30). Delayed virus control in both perforin^−/−^ as well as IFNγ^−/−^ mice did not alter pathology compared to wt mice, indicating that these effector molecules did not play a role in demyelination (34, 35). Similarly, enhanced OLG infection in the absence of IFNγR signaling in OLG did not result in increased demyelination even in the presence of intact T cell function (36). These studies gave the first indication that IFNγ signaling in OLG, independent of their virus load, does not directly affect demyelination.

The role of IFNγ in demyelination nevertheless still remains unresolved. T cell transfer studies with select virus primed T cell populations further indicate that the source of IFNγ in CD4^+^ or CD8^+^ T cells influences pathogenesis. Less demyelination after transfer of IFNγ^−/−^ CD8^+^ T cells into RAG^−/−^ mice correlated with decreased macrophage/microglia activation and recruitment into white matter areas (40). By contrast, transfer of IFNγ^−/−^ CD4^+^ T cells into RAG^−/−^ mice correlated with increased demyelination and mortality (41). The dichotomy of enhanced demyelination in RAG^−/−^ recipient of IFNγ^−/−^ CD4^+^ T cells, which also exhibit selectively increased OLG infection, is likely due to increased IFNγ-regulated neutrophil infiltration and induction of pathogenic Th17 cells (42–44), which had not been uncovered at the time. Distinct from the later studies, lack of IFNγ production by CD4^+^ T cells partially protected SCID recipients from myelin loss, but led to premature mortality (17). Decreased demyelination in SCID recipients of IFNγ^−/−^ CD4^+^ T cells nevertheless also correlated with reduced macrophage infiltration and microglia activation. A direct toxic effect of CD4^+^ T cells on OLG is unlikely due to their lack of MHC class II expression. Some inconsistencies between results in RAG^−/−^ vs. SCID recipients remain to be resolved and may reside in different genetic backgrounds or activation state of transferred T cells (17, 41). Irrespectively, together these data indicate that while IFNγ is vital to reduce MHV virus load, the side effect of extensive macrophages/microglia activation promotes myelin destruction. On the other hand, the total absence of IFNγ not only enhanced virus load, but also maintained neutrophil function and activated Th17 cells (44), which normally do not play a role during a strongly Th1 skewed response during neurotropic MHV infection. More in depth analysis of the role of IFNγ, specifically its cellular targets, is expected to reveal a better understanding of IFNγ as a major regulator of inflammation by promoting MHC class II and iNOS expression and shaping the composition of CNS inflammatory response by regulating chemokine expression. Although iNOS upregulation and oxidative damage have been implicated as factors contributing to CNS tissue damage during demyelination (45), neither genetic ablation of iNOS or pharmacological inhibition of NO affected viral control, demyelination or mortality following infection with v2.2-1 or the neuro attenuated MHV-OBLV60 (46, 47). By contrast, compounds reducing reactive oxygen species (ROS) reduced neuronal loss and demyelination during MHV-A59 induced optic neuritis (48). The contribution of ROS to pathogenesis thus requires more in depth analysis.

## IL-10: the Gamekeeper of Tissue Damage During Chronic JHMV Infection

Incomplete control of neurotropic MHV results in persistent infection characterized by low levels of viral RNA in spinal cord, sustained detection of cytokine and chemokine expression, retention of CD4^+^ and CD8^+^ T cells and ongoing primary demyelination balanced by remyelination (6, 7, 11). The inability to completely eliminate virus suggested an important host response to dampen myelin loss at the expense of virus persistence. One checkpoint molecule was the T cell inhibitory molecule B7-H1, strongly upregulated on OLG. The severity of tissue destruction within lesions in the absence of B7-H1 coincided with increased mortality, although viral control was accelerated (37). Another molecule counteracting tissue damage is the anti-inflammatory cytokine IL-10, known to be a master regulator of immunity to infection (49) as well as balancing immune responses and neurodegeneration in the brain (50). IL-10 is upregulated during acute v2.2-1 infection, at which time it is mainly produced by CD4^+^ and to a lesser extent CD8^+^ T cells (51). While IL-10 expression by CD8^+^ T cells wanes during persistence, it is maintained by CD4^+^ T cells (52, 53). Both Foxp3 regulatory CD4^+^ T cells (Tregs) and virus-specific IFNγ^+^IL-10^+^ CD4^+^ T cells (Tr1) are sources of IL-10 throughout the course of JHMV infection and their role have been recently reviewed by Perlman et al. (54). V2.2-1 infection of IL-10^−/−^ mice resulted in faster control of virus replication during acute infection and reduced initial demyelination; surprisingly however, the severity of demyelination increased 2 weeks after viral control without altering viral persistence (55). IL-10 deficiency was also associated with sustained MHC class II expression on Iba1^+^ myeloid cells and increased iNOS levels in lesions. These data suggested a critical role of IL-10 in limiting tissue damage, despite similar levels of persisting virus. Increased IL-10 production following CNS infection using an engineered IL-10 expressing v2.2-1 variant also resulted in decreased demyelination while virus clearance was slightly delayed (56).

The confirmation of IL-10 as a critical regulator of demyelination questioned whether Tr1 and Foxp3 Tregs played a distinct role. As IL-10 induction in Tr1 cells is IL-27-dependent, mice deficient in IL-27 signaling (IL-27R^−/−^) infected with v2.2-1 were analyzed for a role of Tr1 cells (57). Infected IL-27R^−/−^ displayed drastically reduced Tr1 cells as anticipated, and significantly reduced IL-10 levels at d7 p.i. consistent with faster viral control, similar to IL-10^−/−^ mice. However, impaired IL-27R signaling also correlated with decreased demyelination distinct from the IL-10^−/−^ infected mice. While these findings implied that IL-10 mediated suppression of demyelination is Tr1-independent, it is noted that IL-27R^−/−^ mice have several other dysregulated immune pathways (58, 59). Switching the focus on Foxp3 Tregs, transfer of naïve Foxp3 Tregs into wt or RAG1^−/−^ recipients during acute infection ameliorated tissue damage without affecting virus control (52, 60). These results from a gain of function approach were supported by depletion of CD25^+^ Tregs prior to infection, which resulted in increased demyelination (57). While the effect of Foxp3 Tregs on tissue damage is manifested during chronic infection, their regulatory function may already be initiated during acute infection. Indeed, depletion of Foxp3 Tregs during chronic infection had no effect on the extent of myelin loss (61). Similarly, IL-10 neutralization coincident with CNS infection induced increased demyelination whereas delayed IL-10 inhibition did not affect tissue damage (56). Lastly, although Foxp3 Treg transfer during acute infection decreased CNS tissue damage, they were not detected within the CNS. They rather exerted their functions within CNS draining cervical lymph nodes (CLN) by dampening dendritic cell activation and T cell proliferation (60). These data are consistent with a critical regulatory role of Foxp3 Tregs at the time of initial T cell activation with remote consequences on tissue damage.

Irrespective of Treg effects on effector T cells, increased demyelination in IL-10^−/−^ mice correlated with sustained microglia activation and impaired glial scar formation (55). These results supported a local regulatory role of IL-10 acting directly on CNS resident cells. The downregulation of IL-10Rα expression on microglia, yet upregulation on lesion associated astrocytes further highlights the complex dynamics of the CNS environment in responding to IL-10 (55). The identity of the Foxp3 Treg population limiting tissue damage also requires further investigation. A small population of virus-specific Foxp3 Tregs was detected in both CLN and CNS, where they effectively regulated the pro-inflammatory T cell response at both sites (62). Whether these virus-specific Foxp3 Tregs also play a role in directly regulating demyelination remains to be ascertained. Foxp3 Tregs may also prevent tissue damage during chronic MHV infection by limiting the autoimmune response (63). Global Foxp3 Treg depletion during acute infection correlated with increased proliferation of transferred self-reactive T cells within both CLN and CNS (64). A correlation with potential expansion of demyelinated lesions was however not evaluated. The interplay of various IL-10 secreting Tregs acting at specific sites and on selective target cells at critical time points emphasizes the complex role of IL-10 in dampening JHMV-induced tissue damage without affecting viral clearance and persistence.

Pronounced effects of IL-10 on pathogenesis and clinical outcome rather than viral control in the CNS are also clearly evident in other viral encephalitis models. In the TMEV-mediated transient polioencephalitis model using SJL mice, peak virus load in the hippocampus coincides with peak expression of *IL-10, IL-10ra*, and relates genes. IL-10R neutralization resulted in increased loss of mature neurons and axonal damage, which correlated with enhanced inflammation, although virus load was not altered (65). Further, increased accumulation of Foxp3 Tregs and arginase-1 expressing microglia/macrophages suggested unsuccessful efforts of the host to compensate for the abrogated IL-10 signaling. IL-10 signaling also protects from CNS damage in mice infected with a virulent strain of the mosquito borne alphavirus Sindbis virus by mitigating detrimental Th17 cell functions (66). By contrast, using a more attenuated Sindbis virus, IL-10 deficiency led to longer morbidity, higher mortality, and delayed viral clearance without affecting Th17 cells. Morbidity was rather associated with increased Th1 and decreased Th2 T cells and delayed humoral immunity (67). Along with TNF-α and IL-2, IL-10 is also a key factor for disease remission from fatal encephalitis due to infection with Oshima strain of Tick born encephalitis virus (68). In a murine model of Japanese encephalitis virus infection, elevated IL-10 and reduced IFNγ also correlated with better survival (69). Lastly, IL-10 treatment has been shown to reduce levels of proinflammatory cytokines and infiltrate in murine HSV keratitis without impairing viral clearance (70). *In vivo* results further suggest that IL-10 has the ability to regulate microglial cell production of immune mediators and thereby dampen the pro-inflammatory response to HSV-1 (71).

## Conclusion

**Figure 1:**
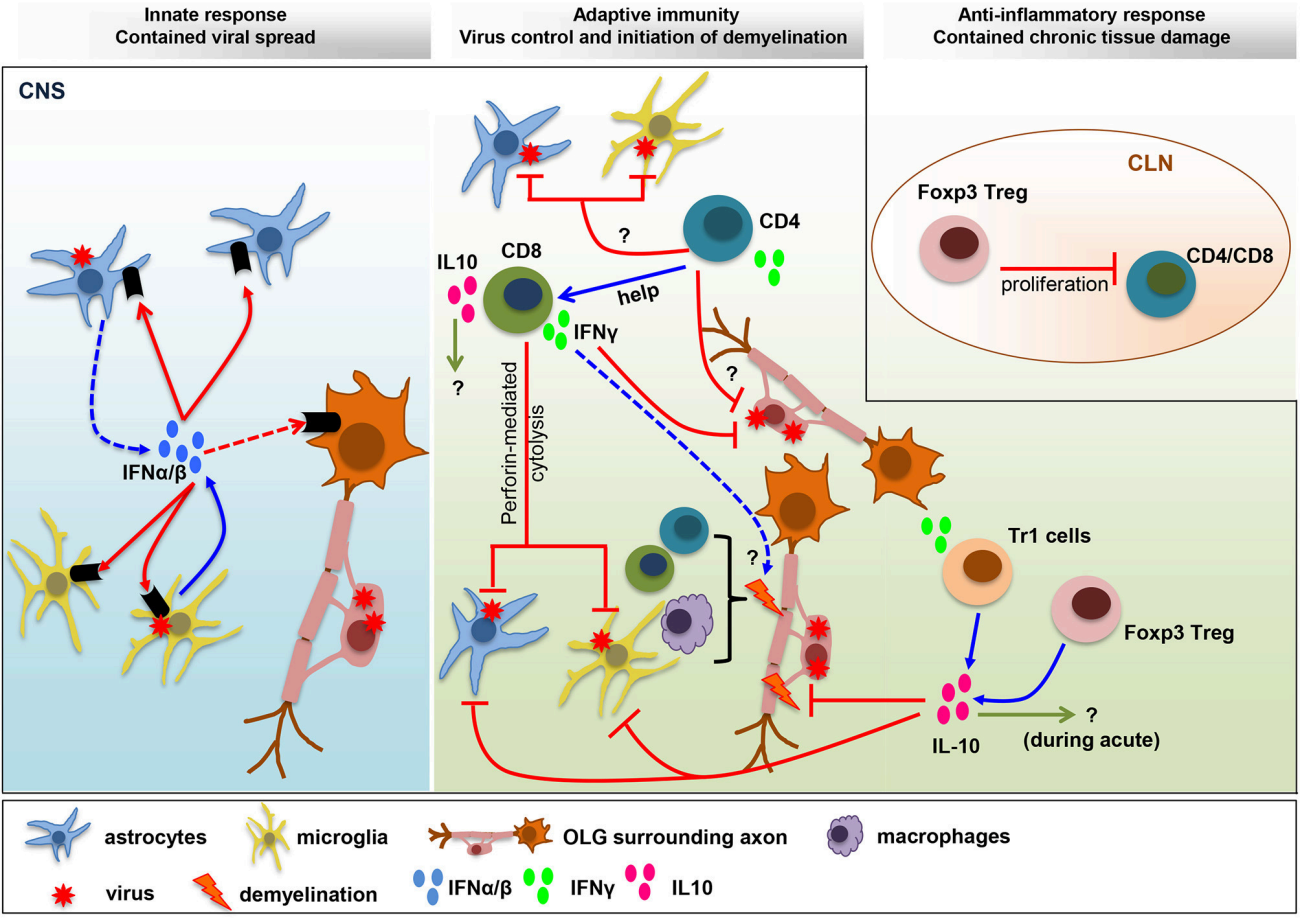
Balance IFN and IL-10 responses determine viral control and pathology. IFNα/β limits viral spread throughout the CNS following MHV infection. The collaboration of microglia as early IFNα/β inducers, and astrocytes as amplifiers of IFNα/β, is crucial to protect from viral dissemination and expanded tropism. The innate response promotes virus-specific T cell recruitment and anti-viral activity critical to eliminate infectious virus below detection limits. CD4^+^ T cells enhance CD8^+^ T cell functions and survival and exhibit uncharacterized anti-viral activity. Virus-specific CD8^+^ T cells eliminate virus using perforin-dependent mechanism in astrocyte/microglia and IFNγ in OLG. CNS T cell recruitment also correlates with initiation of demyelination. Both CD4^+^ and CD8^+^ T cells participate in tissue destruction by instructing myeloid cells to initiate tissue damage. The adverse effects mediated by the pro-inflammatory anti-viral response are balanced by IL-10, a master regulator of immunity to infection. While the role of IL-10 during acute infection remains unknown, it limits myelin loss during chronic infection without affecting viral persistence. Both Foxp3 Tregs and Tr1 cells produce IL-10, which restrain demyelination by regulating microglia activation and astroglial scar formation. A direct role of Foxp3 Treg on peripheral T cell activation, with remote temporal effects on tissue damage, has been suggested by T cell transfer studies.

Animal models of viral CNS infection have been crucial in revealing mechanisms of viral control, establishment of persistence and tissue damage. A common theme, not only applying to neurotropic MHV encephalomyelitis, are the protective activities of IFNα/β signaling in limiting initial viral dissemination and predominantly non-cytolytic T cell effector functions in reducing infectious virus load (1, 2). While some viruses are cytolytic to their target cells, the immune response also actively contributes to bystander damage manifested in glia and neuronal dysfunction or demyelination associated with axonal damage. The neurotropic MHV model specifically highlights the critical role of IFNα/β signaling in a single cell type in stemming overwhelming viral dissemination despite no evident defects in T cell function (Figure 1). It further demonstrates that maximal T cell anti-viral activity during acute infection coincides with maximal anti-inflammatory IL-10 expression, suggesting that an overaggressive adaptive immune response is already counterbalanced during the viral clearance phase, and does not necessarily emerge as a result of tissue damage (Figure 1). Most importantly, the lack of this anti-inflammatory activity can manifest in exacerbated tissue damage remote from acute infection. An immune mediated imbalance early during encephalomyelitis may thus also explain distinct severities of neurological sequelae following human viral disease. For example, IL-6 and IFNγ levels in CSF may be associated with enterovirus (EV)71-induced neuropathology (72). Further, analysis of serum and CSF samples from patients with acute encephalitis syndrome, including with Japanese encephalitis virus supported that higher IL-10 levels in both serum and CSF correlates with protection (73). Similarly, a distinct study of encephalitis patients, including a subcohort with HSV-1, revealed that IL-10 levels were associated with a better coma score on admission in the overall cohort. Elevated IL-10 levels were also associated with a lesser degree of BBB permeability (74). IL-10 signaling also supports BBB integrity following traumatic CNS injury in rodent models (75). With respect to human virus induced encephalitis, it is also interesting to note IL-10 gene polymorphisms as potential susceptibility factors (76). Mutations in IL-10Ra have also been identified as a risk factor of severe influenza-associated encephalopathy (77).

The imprinting of the innate immune response on subsequent adaptive immunity and its effects on bystander cells such as microglia and infiltrating myeloid cells make it difficult to tease apart critical checkpoints determining disease progression or resolution. However, the availability of numerous conditional knockout mice blocking cytokine responses in distinct cell types and in a temporal fashion promise to shed more light on pathways ameliorating pathology while preserving viral control. Confirmation of similar pathways in multiple viral encephalomyelitis models will ultimately enhance targeted treatment options at early stages of disease manifestation. Accumulating literature in both rodent models and human encephalitis implicate that manipulation of IL-10 and IFNγ may have broad implications to treat encephalitis more broadly.

## Author Contributions

CS and CB contributed to the writing, editing of the manuscript and approved the final version for publication.

### Conflict of Interest Statement

The authors declare that the research was conducted in the absence of any commercial or financial relationships that could be construed as a potential conflict of interest.
